# Small-wedge synchrotron and serial XFEL datasets for Cysteinyl leukotriene GPCRs

**DOI:** 10.1038/s41597-020-00729-2

**Published:** 2020-11-12

**Authors:** Egor Marin, Aleksandra Luginina, Anastasiia Gusach, Kirill Kovalev, Sergey Bukhdruker, Polina Khorn, Vitaly Polovinkin, Elizaveta Lyapina, Andrey Rogachev, Valentin Gordeliy, Alexey Mishin, Vadim Cherezov, Valentin Borshchevskiy

**Affiliations:** 1 Research Сenter for Molecular Mechanisms of Aging and Age-related Diseases, Moscow Institute of Physics and Technology, Dolgoprudny, 141701 Russia; 2 Institut de Biologie Structurale (IBS), Université Grenoble Alpes, CEA, CNRS, Grenoble, France; 3 Institute of Biological Information Processing (IBI-7: Structural Biochemistry), Forschungszentrum Jülich GmbH, 52425 Jülich, Germany; 4 Institute of Crystallography, University of Aachen (RWTH), Aachen, Germany; 5 Bridge Institute, Michelson Center for Convergent Bioscience, University of Southern California, Los Angeles, CA 90089 USA; 6 Department of Chemistry, University of Southern California, Los Angeles, CA 90089 USA; 7 JuStruct: Jülich Center for Structural Biology, Forschungszentrum Jülich GmbH, 52425 Jülich, Germany; 8 Joint Institute for Nuclear Research, Dubna, 141980 Russia; 9 ELI Beamlines, Institute of Physics, Czech Academy of Science, Na Slovance 2, 18221 Prague, Czech Republic; 10 Present Address: MRC Laboratory of Molecular Biology, Cambridge CB2 0QH, UK; 11 ESRF—The European Synchrotron, 38000 Grenoble, France

**Keywords:** Data publication and archiving, Nanocrystallography

## Abstract

Structural studies of challenging targets such as G protein-coupled receptors (GPCRs) have accelerated during the last several years due to the development of new approaches, including small-wedge and serial crystallography. Here, we describe the deposition of seven datasets consisting of X-ray diffraction images acquired from lipidic cubic phase (LCP) grown microcrystals of two human GPCRs, Cysteinyl leukotriene receptors 1 and 2 (CysLT_1_R and CysLT_2_R), in complex with various antagonists. Five datasets were collected using small-wedge synchrotron crystallography (SWSX) at the European Synchrotron Radiation Facility with multiple crystals under cryo-conditions. Two datasets were collected using X-ray free electron laser (XFEL) serial femtosecond crystallography (SFX) at the Linac Coherent Light Source, with microcrystals delivered at room temperature into the beam within LCP matrix by a viscous media microextrusion injector. All seven datasets have been deposited in the open-access databases Zenodo and CXIDB. Here, we describe sample preparation and annotate crystallization conditions for each partial and full datasets. We also document full processing pipelines and provide wrapper scripts for SWSX and SFX data processing.

## Abstract (summary)

**Table:** 

Measurement(s)	X-ray diffraction data • protein complex • protein structure data • protein crystallization
Technology Type(s)	small-wedge synchrotron crystallography • x-ray crystallography assay • X-ray free electron laser serial femtosecond crystallography
Factor Type(s)	type of G-protein-coupled receptor • type of antagonist

Machine-accessible metadata file describing the reported data: 10.6084/m9.figshare.13128758

## Background & Summary

Cysteinyl leukotrienes, produced from arachidonic acid via the 5-lipooxygenase pathway, are pro-inflammatory mediators that modulate vascular permeability and immune response; hence, they are involved in multiple disorders including asthma, cardiovascular diseases and cancer^1^. Cysteinyl leukotrienes elicit their action through two G protein-coupled receptors (GPCRs), CysLT_1_R and CysLT_2_R, that share 38% sequence identity^1^. CysLT_1_R is mostly expressed in the lungs and immune cells, and its stimulation leads to allergic symptoms in the airways^2^. CysLT_2_R is found additionally in cardiovascular and brain tissues, with demonstrated involvement in ischemia and acute brain injuries^3,4^, however, the role of this receptor remains controversial and poorly understood. Both CysLT_1_R and CysLT_2_R have been implicated in progression of various cancers^5–8^, while the mutated form of CysLT_2_R with L129Q substitution has been associated with uveal melanoma^9,10^. Thus, CysLTRs are important pharmaceutical targets^11^, what inspired us to determine their high-resolution structures in complex with antiasthmatic drugs and other prospective antagonists.

Over the last few years, small-wedge synchrotron crystallography (SWSX) and serial femtosecond crystallography (SFX) have developed into powerful techniques, enabling high-resolution structure determination of many difficult to crystallize targets^12,13^. Several approaches to data processing have been developed for both SFX^13^ and SWSX^14–20^, and several papers reported deposition of raw serial crystallography data for challenging targets^21–25^. Many datasets can be found online on SBGrid (data.sbgrid.org)^26^, Zenodo (zenodo.org) or CXIDB (cxidb.org)^27^; the latter is used for SFX and other XFEL-related data deposition, whereas SBGrid and Zenodo host SWSX among other types of data.

Recently, we have determined crystal structures of CysLT_1_R^28^ (PDB codes 6RZ4, 6RZ5) and CysLT_2_R^29^ (PDB codes 6RZ6, 6RZ7, 6RZ8, 6RZ9). Here, we present fully-annotated SWSX and SFX datasets for these structures, as well as unpublished SFX data of a new crystal form of CysLT_1_R. The raw diffraction data, consisting of five SWSX and two SFX datasets, represent a wide range of resolutions (2.4–3.5 Å), SWSX miniset^30^ wedge sizes (3–180°), and space groups (6 different space groups). We carefully document crystallization conditions and harvesting details for each dataset, allowing one to investigate crystal non-isomorphism. Finally, we describe all data processing steps, provide supporting code and intermediate results, aiming for reproducibility of deposited data processing.

## Methods

The preparation of CysLT_1_R and CysLT_2_R samples, data collection, and processing have been described previously^28,29^. Here, we provide a summary for each sample.

### Construct engineering, expression, purification, and crystallization of CysLT_1_R and CysLT_2_R

**Fig. 1:**
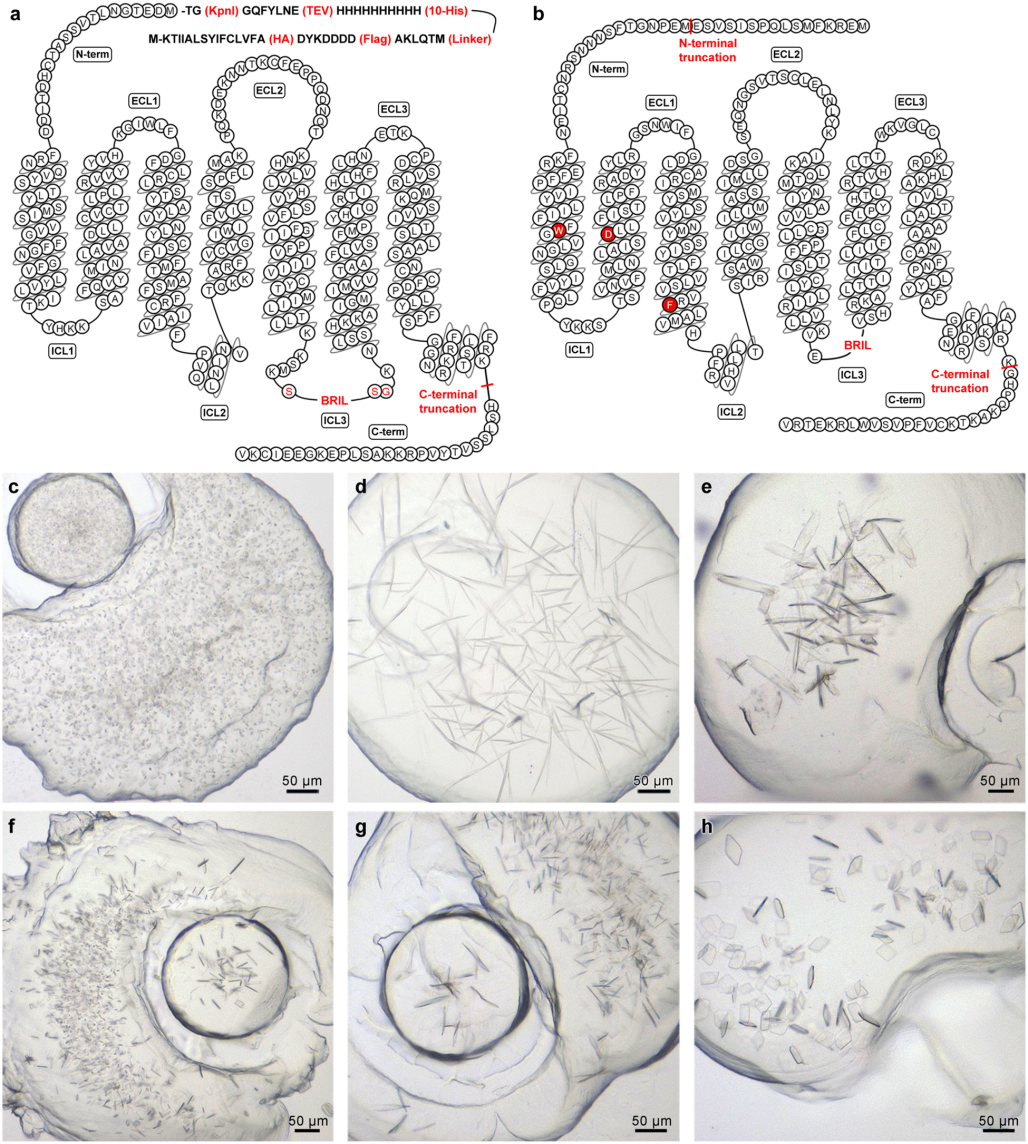
Construct design and crystallization of CysLT_1_R and CysLT_2_R. (**a,b**) Amino acid sequence snake-plot of the CysLT_1_R (*a*) and CysLT_2_R (*b*) crystallization constructs. Protein modifications are shown in red, red background shows stabilising point mutations, red font amino acids – linkers for BRIL insertion. Initial figure drawn using GPCRdb^49^. The N-terminal peptide fragment (*a*) was added to both constructs. (**c**–**h**) microphotographs of typical crystals grown in lipidic cubic phase (LCP) for CysLT_1_R-zafirlukast (*c*), CysLT_1_R-pranlukast (*d*), and CysLT_2_R with antagonists: 11a (C222_1_ space group) (*e*), 11a (F222 space group) (*f*), 11b (*g*), and 11c (*h*) complexes^51^.

The human CysLT_1_R gene (UniProt ID Q9Y271) was codon-optimized for expression in *Spodoptera frugiperda* (*Sf9)* insect cell line and modified for crystallization by a C-terminal truncation at K311 and by the insertion of a fusion protein BRIL^31^ (thermostabilized apocytochrome b_562_ from *Escherichia coli* with mutations M7W, H102I, and R106L) in the third intracellular loop (ICL3) between K222 and K223 using the S and SG linkers on each side, respectively (Fig. 1a). For CysLT_2_R, the human WT gene (UniProt ID Q9NS75) was modified by truncating amino acids 1–16 from the N-terminus and 323–346 from the C-terminus and inserting BRIL into ICL3 between residues E232 and V240. Three point mutations, W51^1.45^V, D84^2.50^N, and F137^3.51^Y (superscripts refer to the generic Ballesteros-Weinstein numbering of residues in Class A GPCR^32^), were further introduced to improve receptor surface expression as well as its stability and yield (Fig. 1b).

Each gene of interest was cloned into a modified *pFastBac1* plasmid, containing a cleavable influenza hemagglutinin signal sequence (HA), a Flag tag, a AKLQTM linker, a 10 × His tag, and a Tobacco Etch Virus (TEV) protease site followed by KpnI restriction site on the N-terminal side of the inserted gene (Fig. 1c). The plasmid was then transfected into *Sf9* insect cells using the bac-to-bac expression system (Invitrogen). High-titer recombinant baculovirus (>3 × 10^8^ viral particles per ml) was obtained and used to infect *Sf9* cells at a density of (2-3) × 10^6^ cells per ml culture and a multiplicity of infection of 5–10 in the presence of a ligand: 8 µM zafirlukast (Cayman Chemical) for CysLT_1_R or 3 µM BayCysLT_2_ (Cayman Chemical) for CysLT_2_R. The protein surface expression and the virus titer were measured using flow cytometry. Cells were harvested 48–50 hours post infection by centrifugation at 2,000 × g and stored at −80 °C until use.

Protein purification was conducted at 4 °C. For each protein-ligand complex, the relevant ligand was added during purification. Cells were thawed and lysed by repetitive homogenization with a glass douncer followed by ultracentrifugation (30 min at 220,000 × g), 2 times in hypotonic buffer (10 mM HEPES pH 7.5, 20 mM KCl, and 10 mM MgCl_2_) and 3 times in high osmotic buffer (10 mM HEPES pH 7.5, 20 mM KCl, 10 mM MgCl_2_, and 1 M NaCl) with the addition of a protease inhibitor cocktail (500 µM 4-(2-aminoethyl)benzenesulfonyl fluoride hydrochloride (Gold Biotechnology), 1 µM E-64 (Cayman Chemical), 1 µM Leupeptin (Cayman Chemical), 150 nM Aprotinin (A.G. Scientific)).

Membranes were then incubated for 30 min in 10 mM HEPES pH 7.5, 20 mM KCl, 10 mM MgCl_2_, 2 mg ml^−1^ iodacetomide, protease inhibitor cocktail, and 25 µM ligand. Then receptors were solubilized by the addition of an equal volume of solubilisation buffer (300 mM NaCl, 2% (w/v) n-dodecyl-β-D-maltopyranoside (DDM; Avanti Polar Lipids) 0.4% (w/v) cholesteryl hemisuccinate (CHS; Sigma), 10% glycerol) and incubation for 3.5 hours. After 1-hour centrifugation (650,000 × g) to remove insolubilized material, the supernatant was incubated with a TALON IMAC resin (Clontech) overnight in the presence of 10/20 mM imidazole, 100 мМ HEPES pH 7.5 for CysLT_1/2_R with NaCl concentration increased to 800 mM.

The resin was then washed with 10 column volumes (CV) of wash buffer I (8 mM ATP, 100 mM HEPES pH 7.5, 10 mM MgCl_2_, 500 mM NaCl, 15 mM imidazole, 10 μM ligand, 10% glycerol, 0.1% DDM, 0.02% CHS), then with 5 CV of wash buffer II (25 mM HEPES pH 7.5, 250/500 mM NaCl for CysLT_1/2_R, 30 mM imidazole, 10 μM ligand, 10% glycerol, 0.015% DDM, 0.003% CHS), then buffer was exchanged into buffer III (25 mM HEPES pH 7.5, 250/500 mM NaCl for CysLT_1/2_R, 10 mM imidazole, 10 μM ligand, 10% glycerol, 0.05% DDM, 0.01% CHS) and the protein-containing resin was treated with PNGase F (Sigma) for 5 hours. Resin was further washed with 5 CV of wash buffer III and eluted with (25 mM HEPES pH 7.5, 250/500 mM NaCl for CysLT_1/2_R, 300 mM imidazole, 10 μM ligand, 10% glycerol, 0.05% DDM, 0.01% CHS) in several fractions. After removing imidazole using a PD10 desalting column (GE Healthcare), the protein was incubated with 50 µM ligand and a His-tagged home-made TEV protease overnight to remove the N-terminal tags. Reverse IMAC was performed on the following day and the protein was concentrated up to 50–70 mg ml^−1^ using a 100 kDa molecular weight cut-off centrifugal concentrator (Millipore). The protein purity was checked by SDS-PAGE, and the protein yield and monodispersity were estimated by analytical size exclusion chromatography (aSEC).

Crystals for SWSX were grown using high-throughput nanovolume LCP crystallization. The purified and concentrated protein solution was combined with a lipid mixture: 90% monoolein (Sigma), 10% cholesterol (Affymetrix) in the ratio of 2:3 v/v and homogenized using a lipid syringe mixer until a transparent gel-like LCP formed^33^. Crystallisation was set up in 96-well glass sandwich LCP plates (Marienfeld), with 40 nL LCP drops and 800 nL precipitant drops, which were pipetted using an NT8-LCP robot (Formulatrix). All LCP manipulations were performed at room temperature (20–23 °C), and plates were incubated and imaged at 22 °C using an automated incubator/imager RockImager 1000 (Formulatrix).

CysLT_1_R-pranlukast crystals had a needle shape (Fig. 1d) and gained their full size after 3-4 weeks; however, the best diffraction was obtained from samples incubated for 2 months. CysLT_2_R crystals grew to their full size within 1–3 weeks. Crystals of CysLT_2_R in complex with ligands 11a and 11b had a shape of an elongated plate with a maximal size up to 150 µm (Fig. 1e–g). In case of CysLT_2_R-11c complex, crystals grew as flat parallelepipeds as long as 30–50 µm in diagonal (Fig. 1h). For the full list of crystallization conditions for crystals used in the data collection see Table 1.

**Table 1:** Summary of crystallization conditions for SWSX datasets.

Dataset	Condition ID	No. minisets	Condition
CysLT1R_6RZ4	1	27	100 mM Tris pH8, 33% PEG400, 160 mM Sodium Nitrate, 1% v/v 1,2-Butanediol
	2	4	100 mM Tris pH8, 34% PEG400, 130 mM Sodium Nitrate
	3	40	100 mM Tris pH8, 34% PEG400, 75 mM Sodium Nitrate
	4	8	100 mM Sodium Citrate pH6, 31% PEG400, 200 mM Lithium Nitrate
	5	23	100 mM Tris pH8, 33% PEG400, 160 mM Sodium Nitrate, 4% v/v 2,5-Hexanediol (mixture of isomers)
	6	23	100 mM Sodium Citrate pH6, 34% PEG400, 400 mM Lithium Nitrate, 10 mM Betaine hydrochloride
	7	13	100 mM Tris pH8, 34% PEG400, 266 mM Sodium Nitrate
	8	5	100 mM Sodium Citrate pH6, 34% PEG400, 400 mM Lithium Nitrate, 3% w/v D-( + )-Glucose monohydrate
	9	2	100 mM Sodium Citrate pH6, 34% PEG400, 400 mM Lithium Nitrate, 100 mM Cesium Chloride
	10	2	100 mM Tris pH8.2, 37% PEG400, 200 mM Sodium Nitrate, 4% v/v 2,5-Hexanediol (mixture of isomers)
	11	16	100 mM Sodium Citrate pH6, 34% PEG400, 400 mM Lithium Nitrate, 4% v/v 2,5-Hexanediol (mixture of isomers)
	12	11	100 mM Tris pH8, 33% PEG400, 160 mM Sodium Nitrate, 10 mM Spermine tetrahydrochloride
	13	7	100 mM Sodium Citrate pH6, 30% PEG400, 200 mM Lithium Nitrate
	14	2	100 mM Sodium Citrate pH6, 30% PEG400, 236 mM Lithium Nitrate
	15	1	100 mM Tris pH8, 33% PEG400, 160 mM Sodium Nitrate, 10 mM Spermidine
	16	2	100 mM Tris pH8, 33% PEG400, 160 mM Sodium Nitrate, 0.5% w/v n-Dodecyl-b-D-maltoside
CysLT2R_6RZ6	1	9	100 mM Ammonium Sulfate, 30% v/v PEG400, 100 mM HEPES pH7.0
	2	6	130 mM Ammonium Tartrate dibasic, 30% v/v PEG400, 100 mM HEPES pH 7.0, 10 mM Spermine tetrahydrochloride
	3	12	100 mM Ammonium Sulfate, 30% v/v PEG400, 100 mM HEPES pH7.0
	4	2	100 mM Lithium Sulfate monohydrate, 30% v/v PEG400, 100 mM HEPES pH7.0
	5	18	30 mM Ammonium Tartrate dibasic, 20% v/v PEG400, 100 mM HEPES pH 7.0, 10 mM Betaine hydrochloride
	6	7	60 mM Ammonium Tartrate dibasic, 22% PEG400, 100 mM HEPES pH7.0
	7	19	70 mM Ammonium Tartrate dibasic, 22% PEG400, 100 mM HEPES pH7.0
	8	2	30 mM Ammonium Tartrate dibasic, 20% PEG400, 100 mM HEPES pH7.0
	9	7	100 mM Ammonium Tartrate dibasic, 30% v/v PEG400, 100 mM TRIS pH8.0
	10	9	100 mM Ammonium Tartrate dibasic, 24% PEG400, 100 mM HEPES pH7.0
	11	1	100 mM Ammonium Tartrate dibasic, 26% PEG400, 100 mM HEPES pH7.0
	12	1	120 mM Ammonium Tartrate dibasic, 26% PEG400, 100 mM HEPES pH7.0
	13	7	120 mM Ammonium Tartrate dibasic, 28% PEG400, 100 mM HEPES pH7.0
	14	4	100 mM Ammonium Tartrate dibasic, 30% v/v PEG400, 100 mM TRIS pH8.0
	15	17	100 mM Ammonium Tartrate dibasic, 30% v/v PEG400, 100 mM TRIS pH8.0
	16	52	80 mM Ammonium Tartrate dibasic, 24% PEG400, 100 mM HEPES pH7.0
	17	5	130 mM Ammonium Tartrate dibasic, 30% v/v PEG400, 100 mM HEPES pH 7.0, 10 mM Yttrium(III) Chloride hexahydrate
	18	20	100 mM Ammonium Tartrate dibasic, 26% PEG400, 100 mM HEPES pH7.0
	19	3	130 mM Ammonium Tartrate dibasic, 26% PEG400, 100 mM HEPES pH7.0
	20	2	130 mM Ammonium Tartrate dibasic, 28% PEG400, 100 mM HEPES pH7.0
	21	1	30 mM Ammonium Tartrate dibasic, 20% v/v PEG400, 100 mM HEPES pH 7.0, 0.025% v/v Dichloromethane
	22	1	90 mM Ammonium Tartrate dibasic, 24% PEG400, 100 mM HEPES pH7.0
	23	6	30 mM Ammonium Tartrate dibasic, 20% v/v PEG400, 100 mM HEPES pH 7.0, 1% v/v 1,2-Butanediol
	24	9	30 mM Ammonium Tartrate dibasic, 20% v/v PEG400, 100 mM HEPES pH 7.0, 4% v/v 1-Propanol
	25	11	130 mM Ammonium Tartrate dibasic, 27.4% PEG400, 100 mM HEPES pH7.0
	26	5	120 mM Ammonium Tartrate dibasic, 26% PEG400, 100 mM HEPES pH7.0
CysLT2R_6RZ7	1	2	130 mM Ammonium Tartrate dibasic, 30% v/v PEG400, 100 mM HEPES pH 7.0, 0.025% v/v Dichloromethane
	2	4	100 mM Ammonium Tartrate dibasic, 30% v/v PEG400, 100 mM TRIS pH8.0
	3	10	100 mM Ammonium Tartrate dibasic, 24% PEG400, 100 mM HEPES pH7.0
	4	9	100 mM Ammonium Sulfate, 30% v/v PEG400, 100 mM HEPES pH7.0
	5	18	30 mM Ammonium Tartrate dibasic, 20% v/v PEG400, 100 mM HEPES pH 7.0, 10 mM Betaine Hydrochloride
	6	9	30 mM Ammonium Tartrate dibasic, 20% v/v PEG400, 100 mM HEPES pH 7.0, 4% v/v 1-Propanol
	7	8	130 mM Ammonium Tartrate dibasic, 30% v/v PEG400, 100 mM HEPES pH 7.0, 10 mM Yttrium(III) Chloride hexahydrate
	8	3	100 mM Lithium Sulfate monohydrate, 30% v/v PEG400, 100 mM HEPES pH7.0
	9	1	120 mM Ammonium Tartrate dibasic, 26% PEG400, 100 mM HEPES pH7.0
	10	6	110 mM Ammonium Tartrate dibasic, 26% PEG400, 100 mM HEPES pH7.0
	11	1	30 mM Ammonium Tartrate dibasic, 20% v/v PEG400, 100 mM HEPES pH 7.0, 0.025% v/v Dichloromethane
	12	16	120 mM Ammonium Tartrate dibasic, 28% PEG400, 100 mM HEPES pH7.0
	13	14	130 mM Ammonium Tartrate dibasic, 28% PEG400, 100 mM HEPES pH7.0
	14	6	30 mM Ammonium Tartrate dibasic, 20% v/v PEG400, 100 mM HEPES pH 7.0, 1% v/v 1,2-Butanediol
	15	16	30 mM Ammonium Tartrate dibasic, 20% PEG400, 100 mM HEPES pH7.0
	16	23	130 mM Ammonium Tartrate dibasic, 27.4% PEG400, 100 mM HEPES pH7.0
	17	6	100 mM Ammonium Tartrate dibasic, 30% v/v PEG400, 100 mM TRIS pH8.0
	18	6	130 mM Ammonium Tartrate dibasic, 30% v/v PEG400, 100 mM HEPES pH 7.0, 10 mM Spermine tetrahydrochloride
CysLT2R_6RZ8	1	11	100 mM Potassium fluoride, 30% v/v PEG400, 100 mM TRIS pH8.0
CysLT2R_6RZ9	1	18	210 mM Ammonium Tartrate dibasic, 30.9% PEG400, 100 mM HEPES pH7.0
	2	4	100 mM Ammonium Tartrate dibasic, 30% v/v PEG400, 100 mM TRIS pH8.0
	3	3	80 mM Ammonium Tartrate dibasic, 29.14% PEG400, 100 mM HEPES pH7.0
	4	5	20 mM Ammonium Tartrate dibasic, 32.6% PEG400, 100 mM HEPES pH7.0
	6	8	250 mM Ammonium Tartrate dibasic, 32.6% PEG400, 100 mM HEPES pH7.0
	7	2	130 mM Ammonium acetate, 27.4% PEG400, 100 mM HEPES pH7.0
	8	2	50 mM Ammonium Tartrate dibasic, 34.9% PEG400, 100 mM HEPES pH7.0
	9	2	90 mM Ammonium Tartrate dibasic, 29.14% PEG400, 100 mM HEPES pH7.0

Microcrystals of the CysLT_1_R-zafirlukast complex for SFX were grown in 100 µl gas-tight Hamilton syringes as previously described^34,35^. Briefly, approximately 5 µl of protein-laden LCP was transferred through a coupler (Formulatrix) into a syringe, containing 50 µl of precipitant, so that LCP extends towards the plunger as a straight filament. For experiments conducted in 2016 (dataset CysLT1R_zafirlukast-P21), zafirlukast was added at 50 μM prior to the protein concentration. Crystals grew in the following precipitant conditions: 100 mM ammonium phosphate, 31–34% v/v PEG400, 100 mM HEPES pH 7.0, 1 µM zafirlukast. For experiments conducted in 2017 (dataset CysLT1R_6RZ5), zafirlukast was added at 200 μM prior to the protein concentration. Crystals grew in the following precipitant conditions: 120–200 mM sodium/potassium phosphate, 31–34% v/v PEG400, 100 mM HEPES pH 7, no zafirlukast added. Crystals grew for 1-2 weeks, reaching an average crystal size of 5 × 2 × 2 µm (Fig. 1c).

### Synchrotron data collection

#### Crystal harvesting

Crystals were harvested directly from LCP using 50–200 µm dual thickness MicroMounts or 400–700 µm MicroMesh loops (MiTeGen) with various hole sizes and flash frozen in liquid nitrogen, as described^36^.

#### Full sets data collection

Single-crystal datasets (for CysLT1R_6RZ4 and CysLT2R_6RZ8) were collected using the following procedure. First, the best diffracting position was found using automatic X-ray centring^37^ with a microfocus beam, followed by characterization^37^ and dose estimation using BEST^38^ software, and further data collection as proposed by BEST. This resulted in over 90% complete datasets, however, with a relatively low resolution (>3 Å).

#### Partial sets data collection

**Fig. 2:**
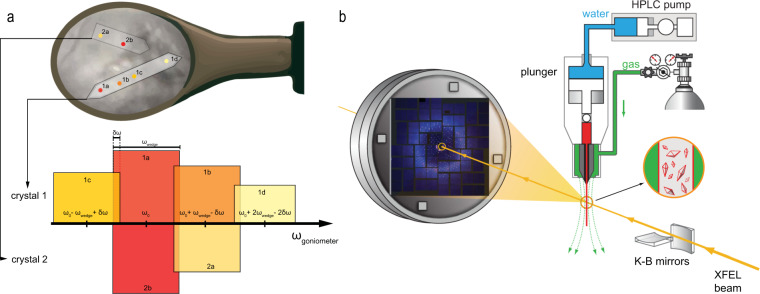
Synchrotron and XFEL data collection setups. (**a**) Schematics of the SWSX data collection process. The bar colour indicates the DOZOR score (from red – the best diffraction, to yellow – the worst). For minisets collected from the same crystal, as judged by the diffraction patterns and DOZOR score heatmap, an overlap of δω = 1–2° is introduced between consecutive sets. When the rotation angle ω exceeds ~10° from the initial orientation, as for the point 1d, an additional line scan is performed to re-align the crystal. The orientations of two different crystals 1 and 2 in the loop are assumed independent, and thus minisets from them are collected within the same ω range. (**b**) LCP-SFX data collection scheme. Microcrystals embedded in LCP are injected inside a vacuum chamber into the XFEL beam focus region using a viscous media microextrusion injector. A stream of sheath gas (nitrogen or helium) is used to keep the LCP stream straight. Microcrystals intersect with the XFEL beam in random orientations and diffraction patterns are collected by a CSPAD detector.

To improve resolution, SWSX partial datasets (minisets, as introduced by Basu *et al*.^30^) were collected using an updated version of the raster-scanning approach^39^. The process is illustrated in Fig. 2a. Each loop was first visually aligned and oriented with its plane perpendicular to the X-ray beam. Then, the whole loop was scanned with the beam to identify locations with diffracting crystals (shutterless mode was used on the ID29 and ID30b beamlines). Raster scans were performed using a minimal dose per image, which allowed for visual detection of diffraction spots, but was less than 1% of the total dose per dataset. The grid spacing was set around $\sqrt{{\bf{2}}}{\boldsymbol{R}}$, where R is the beam profile radius (HWHM). The overlap between adjacent beam spots was introduced to improve accuracy in location of the best diffracting positions and to maximize the grid coverage by HWHM profiles. The grid cells showing diffraction spots were ranked by the DOZOR score^37^ and then manually selected for further data collection. In the case of large single crystals spanning through several grid cells, minisets were collected starting from the highest ranked location and then moving to the next best location along the crystal but skipping grid cells if they had a common edge with the cells already used for data collection to avoid collecting data from previously exposed parts of the crystal. Consecutive minisets from the same crystal were collected by ensuring 1-2° overlaps in the goniometer rotation ω angle. When the goniometer rotation angle exceeded 10° from its original orientation, a new line raster scan was performed to re-align the crystal with the beam. Each miniset was collected restricting an estimated dose per diffraction location within ∼20 MGy and using 0.1–0.2° oscillation and 3–20° total wedge size. The wedge size and the corresponding exposure time were selected based on the total number of harvested crystals from the particular condition and were adjusted by decreasing the wedge size and increasing the exposure time when preliminary data processing indicated that a complete dataset had been already collected, or in case of a weak diffraction. The beam size was chosen to match the smallest crystal dimension. A summary of miniset parameters for each SWSX entry is given in Table 2.

**Table 2:** Summary of SWSX datasets.

Entry	CysLT1R_6RZ4	CysLT2R_6RZ6	CysLT2R_6RZ7	CysLT2R_6RZ9	CysLT2R_6RZ8
Reported resolution, Å	2.7	2.43	2.43	2.73	2.7
Reprocessed resolution, Å	2.6	2.43	2.43	2.73	2.5
No. collected minisets	186	236	158	44	11
No. processed minisets	116	126	144	38	11
No. minisets in the final dataset	9	74	16	12	8
Wedge size, °	10–180	3–12	4–20	10–20	10–20
Detector/beamline	Eiger-4M/ID30a3 Pilatus3-S-6M/ID30b Pilatus-6M/ID29	Pilatus3-S-6M/ID30b Pilatus-6M/ID29	Eiger-4M/ID30a3 Pilatus-6M/ID23-1 Pilatus-6M/ID29	Pilatus-6M/ID23-1 Pilatus-6M/ID29	Pilatus-6M/ID23-1

### XFEL data collection

#### Loading crystals into injector

Precipitant solutions were slowly withdrawn from 3 syringes containing microcrystals of appropriate size and density through a 22 s gauge Hamilton needle. The remaining samples of LCP with microcrystals embedded in it were consolidated from these 3 syringes into one syringe using a syringe coupler (Formulatrix). An aliquot of ~10% of 7.9 MAG lipid was added to the sample to absorb the excess of the precipitant and to avoid LCP freezing upon extrusion in the vacuum chamber^40^. A total sample volume of 15–20 µl was loaded into an LCP injector as described^40^.

#### LCLS data collection: 2016

An overall scheme of the data collection setup is shown in Fig. 2b. SFX data of CysLT1R_Zafirlukast-P21 were collected in August 2016 at the CXI instrument of the Linac Coherent Light Source (LCLS) at the SLAC National Accelerator Laboratory, Menlo Park, California. LCLS was operated at a wavelength of 1.305 Å (9.50 keV) delivering individual X-ray pulses of 40 fs duration and 2.6 × 10^10^ photons per pulse focused into a spot size of ~1.5 µm in diameter using a pair of Kirkpatrick-Baez mirrors. LCP with protein microcrystals was extruded at room temperature and at a flow rate of 0.3 μl min^−1^ inside a vacuum chamber into the beam focus region using an LCP injector^40^ with a 50-μm diameter capillary. The XFEL beam was attenuated at transmission levels of 6.1% to avoid disruptions of the LCP stream. Diffraction images were collected at an XFEL pulse repetition rate of 120 Hz using a 2.3 Megapixel Cornell-SLAC Pixel Array Detector^41^ (CSPAD).

A total number of 900,173 detector images were collected, of which 22,047 (2% of total) were identified as potential crystal hits with more than 15 Bragg peaks with SNR = 6.0, threshold 100 and min-pix-count 3.0 using peakfinder8 algorithm as implemented in Cheetah^42^. The overall time of data collection from a sample with a total volume of 27 μl was about 2 h 6 min.

#### LCLS data collection: 2017

SFX data of CysLT1R_6RZ5 were collected in August 2017 at the CXI instrument. LCLS was operated at a wavelength of 1.302 Å (9.52 keV) delivering individual X-ray pulses of 43 fs duration and 1.9 × 10^10^ photons per pulse focused into a spot size of ~1.5 µm in diameter using a pair of Kirkpatrick-Baez mirrors. LCP with protein microcrystals was extruded at room temperature and at a flow rate of 0.3 μl min^−1^ inside a vacuum chamber into the beam focus region using an LCP injector^40^ with a 50-μm diameter capillary. The XFEL beam was attenuated at transmission levels of 6.3–10% to avoid disruptions of the LCP stream. Diffraction images were collected at an XFEL pulse repetition rate of 120 Hz using a 2.3 Megapixel Cornell-SLAC Pixel Array Detector^43^ (CSPAD).

A total number of 390,442 detector images were collected, of which 43,417 (11% of total) were identified as potential crystal hits with more than 20 Bragg peaks with SNR = 4.0, threshold 200 and min-pix-count 3.0 using peakfinder8 algorithm as implemented in Cheetah^42^. The overall time of data collection from a sample with a total volume of 15 μl was about 54 min.

#### Data processing

All datasets, except for the SFX dataset CysLT1R_Zafirlukast-P21 (P21 space group), have been previously indexed, integrated, sorted, and merged to solve the structures of the corresponding receptor complexes by molecular replacement, as described^28,29^. Re-processing of the data with the same or better processing statistics as in the original manuscripts is described in the *Technical validation* section.

## Data Records

SWSX data^44–49^ have been deposited to Zenodo under accession numbers provided in Table 3. Each SWSX dataset folder contains subfolders, representing each miniset collected, regardless of the angular range for data collection. Each miniset subfolder is named as XXX_YY_ZZ_NN, where XXX is the sequential number of the miniset, YY is the crystallization condition ID, ZZ is the serial number of the harvesting loop within each crystallization condition, NN – the serial number of the miniset within each loop. Each miniset subfolder contains a subfolder ‘images‘ with all diffraction images in either cbf or HDF5 format. It also contains an XDS parameter file XDS.INP with the keyword NAME_TEMPLATE_OF_DATA_FRAMES pointing to files in ‘images‘ subfolder, and other parameters as used during reprocessing (see keywords for express.py below). Also, each miniset subfolder contains all XDS-related files (including geometry correction x_geo_corr.cbf and y_geo_corr.cbf for cbf files) for this miniset (everything up to CORRECT.LP and XDS_ASCII.HKL for successfully integrated datasets, and only COLSPOT.LP for non-successful ones). A summary of all SWSX entries is shown in Table 2, and a summary of all SWSX entries crystallization conditions is present in Table 1, with a full description provided in Supplementary Table 1.

**Table 3:** Data availability on Internet. Github gist, associated with the publication, has the ‘download_all.sh‘ script for Linux to download all data entries described in this publication.

Entry	Reported resolution in PDB, Å	No. minisets (SWSX) or No. indexed lattices (SFX)	Entry page	Entry size
CysLT2R_6RZ6	2.43	236 (SWSX)	https://zenodo.org/record/4032836	48.2 GB
CysLT2R_6RZ7	2.43	158 (SWSX)	https://zenodo.org/record/4032837	88.1 GB
CysLT2R_6RZ9	2.73	11 (SWSX)	https://zenodo.org/record/4032841	18.7 GB
CysLT2R_ 6RZ8	2.70	44 (SWSX)	https://zenodo.org/record/4032840	8.9 GB
CysLT1R_ 6RZ4	2.70	186 (SWSX)	https://zenodo.org/record/4032826	90.0 GB
CysLT1R_ 6RZ5	2.53	43,417 (SFX)	https://www.cxidb.org/id-106.html	134.9 GB
CysLT1R_Zafirlukast-P21	N/A	22,047 (SFX)	https://www.cxidb.org/id-107.html	193.6 GB

SFX data have been deposited to CXIDB as ID106^50^ (CysLT1R_6RZ5) and ID107^51^ (CysLT1R_Zafirlukast-P21). Only those images identified as crystal hits by Cheetah are included in the deposited dataset. Each SFX dataset folder contains a subfolder ‘raw_data’ with all runs as written by Cheetah, their respective cheetah.ini files and cxi files with images. Also, each SFX dataset folder contains a file ‘initial.geom’ that was used during reprocessing. A summary for all SFX entries is given in Table 4.

**Table 4:** Summary of SFX datasets.

Entry	CysLT1R_6RZ5	CysLT1R_Zafirlukast-P21
Reported resolution, Å	2.53	N/A
Reprocessed resolution, Å	2.50	3.5
Total No. images	390,442	900,173
No crystal hits	43,417	22,047
No. indexed images	28,528	17,193
No. indexed lattices	28,900	17,193

## Technical Validation

### Data processing

During the preparation of this manuscript, all data were re-processed in a consistent manner. Here we present a pipeline for data processing that results in similar or better resolution values and figures of merit compared to those reported in the original papers. Data processing statistics for all datasets is shown in Table 5.

**Table 5:** Crystallographic data collection statistics.

PDB ID	CysLT1R			CysLT2R			
	6RZ4	6RZ5	Zafirlukast-P21	6RZ6	6RZ7	6RZ9	6RZ8
Dataset type	SWSX	SFX	SFX	SWSX	SWSX	SWSX	SWSX
No. crystals (SFX) or No. minisets (SWSX)	9	28,900	17,193	74	16	12	8
Cell dimensions							
*a b c*, Å	59.39 45.89 87.13	41.58 68.56 87.37	43.86 159.43 73.48	69.34 167.95 85.44	81.82 142.07 171.21	67.67 165.01 82.66	78.40 78.40 171.91
α β γ, °	90.00 95.69 90.00	76.33 76.69 81.19	90.00 95.12 90.00	90.00 90.00 90.00	90.00 90.00 90.00	90.00 90.00 90.00	90.00 90.00 90.00
Space group	P2_1_	P1	P2_1_	C222_1_	F222	I4	C222_1_
Resolution, Å#	30.0-2.6 (2.67-2.60)	30.0-2.53 (2.57-2.53)	30.0-3.5 (3.56-3.50)	30.0-2.3 (2.36-2.30)	30.0-2.43 (2.49-2.43)	30.0-2.5 (2.56-2.50)	30.0-2.73 (2.80-2.73)
Completeness, %^#^	99.9 (100.0)	100.0 (100.0)	100.0 (100.0)	99.2 (97.6)	98.2 (98.0)	99.9 (100.0)	94.7 (95.4)
Redundancy^#^	7.24 (6.86)	67.6 (31.0)	128.7 (73.6)	7.39 (5.80)	6.15 (6.25)	12.5 (12.8)	3.27 (3.35)
R_meas_ (SWSX) or R_split_ (SFX)^#^	0.35 (1.05)	0.23 (2.80)	0.16 (2.59)	0.30 (2.74)	0.31 (3.33)	0.24 (5.52)	0.23 (3.06)
I/σ(I)^#^	5.59 (1.93)	2.89 (0.42)	4.17 (0.46)	5.75 (0.84)	5.48 (0.79)	8.62 (0.67)	3.72 (0.69)
CC*^#^	1.00 (0.57)	0.99 (0.51)	1.00 (0.61)	1.00 (0.65)	1.00 (0.63)	1.00 (0.50)	1.00 (0.57)

^#^Values in parentheses are for highest-resolution shell.

### SWSX data

**Fig. 3:**
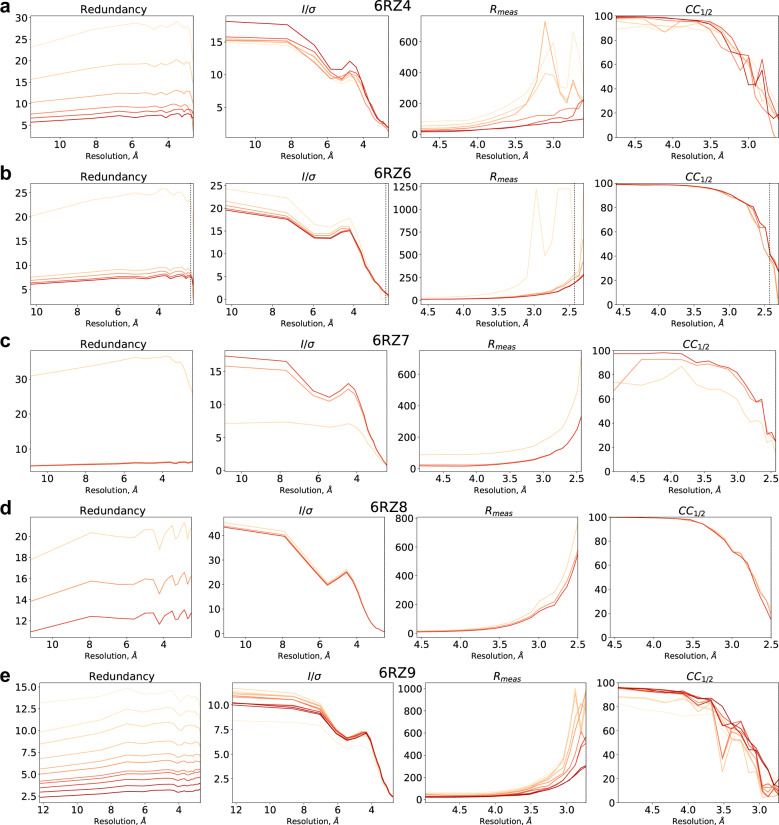
Improvements in data merging statistics for SWSX datasets during ΔCC_1/2_ rejection process. (**a**–**e**) Plots of redundancy, I/σ, R_meas_ and CC_1/2_ vs resolution for each data processing iteration are shown for the following datasets: CysLT1R_6RZ4 (*a*), CysLT2R_6RZ6 (*b*), CysLT2R_6RZ7 (*c*), CysLT2R_6RZ8 (*d*), CysLT2R_6RZ9 (*e*). Darker curves represent latter stages of ΔCC_1/2_ miniset rejection.

For SWSX data, the processing algorithm works as following (note that the treatment of both full datasets and minisets is the same). For each dataset, initial indexing and integration are performed by XDS within the resolution range of 40–2.5 Å using the beamline-provided XDS.INP file, without specifying the unit cell parameters and the space group identity (for Dectris images, the “neggia” library was used, as described here (https://strucbio.biologie.uni-konstanz.de/xdswiki/index.php/Eiger). Each integration runs first with the keywords “JOB = XYCORR INIT COLSPOT IDXREF DEFPIX INTEGRATE CORRECT”, then the integration parameters are updated using the output of the CORRECT step as described in the section “Final polishing: Re-INTEGRATEing with the correct spacegroup, refined geometry and fine-slicing of profiles” (https://strucbio.biologie.uni-konstanz.de/xdswiki/index.php/Optimisation#Re-INTEGRATEing_with_the_correct_spacegroup.2C_refined_geometry_and_fine-slicing_of_profiles), and integration is re-run using the keywords “JOB = DEFPIX INTEGRATE CORRECT”. After that, the algorithm attempts to scale all obtained XDS_ASCII.HKL files using XSCALE, and runs several rounds of ΔCC_1/2_ rejection of non-isomorphous minisets using xdscc12 subprogram as described^17^ (until there are no rejected minisets at the subsequent iteration). For most datasets, the miniset rejection was applied in two steps: first, using the low-resolution range (e.g. 30.0–10.0 Å, ΔCC_1/2_ threshold 0–2), and then using the high-resolution range (e.g. 10.0-2.5 Å, ΔCC_1/2_ threshold 1–5). All processing parameters are summarized in Table 6. The obtained dataset is merged and used as a REFERENCE_DATA_SET during the 2^nd^ integration attempt of all minisets (including those rejected during the previous integration attempts). If CC_1/2_ in the highest resolution shell exceeds 0.15, the RESOLUTION_RANGE is increased manually for the 3^rd^ integration. Next, another round of ΔCC_1/2_ rejection of non-isomorphous minisets is performed followed by merging to produce a final dataset. Improvements in the figures of merit for a dataset as a result of ΔCC_1/2_ rejection are shown in Fig. 3. For the CysLT2R_6RZ8 dataset (space group I4), there is an indexing ambiguity with two indexing options available for each miniset, thereby some minisets have to be re-indexed using ‘REIDX_ISET = 0 -1 0 0 -1 0 0 0 0 0 -1 0‘ keyword in XSCALE. This is done by following an iterative procedure: first, two largest minisets are merged together using two possible indexing options for the second set, and the indexing option resulting in a smaller R_meas_ is chosen. Then, all other minisets are added one by one, using the indexing choice that producess smaller R_meas_ for the merged dataset. For the final merged dataset, phenix.xtriage reports no significant twinning.

**Table 6:** SWSX data processing parameters. For CysLT2R_6RZ6 and CysLT2R_6RZ7, only full resolution range rejection was performed.

Entry		CysLT2R_6RZ6	CysLT2R_6RZ7	CysLT2R_6RZ9	CysLT2R_6RZ8	CysLT1R_6RZ4
Low-resolution rejection	Resolution limits, Å/No. bins	N/A	N/A	30.0-10.0/17	30.0-10.0/7	32.0-10.0/5
	ΔCC_1/2_ threshold	N/A	N/A	0.0	1.0	2.0
	No. iterations	0	0	5	5	4
High-resolution rejection	Resolution limits, Å/No. bins	30.0-2.3/23	30.0-2.43/23	30.0-2.73/17	10.0-2.5/15	5.0-2.6/15
	ΔCC_1/2_ threshold	3.0	3.0	5.0	1.0	2.0
	No. iterations	5	5	7	4	5

### SFX data

**Fig. 4:**
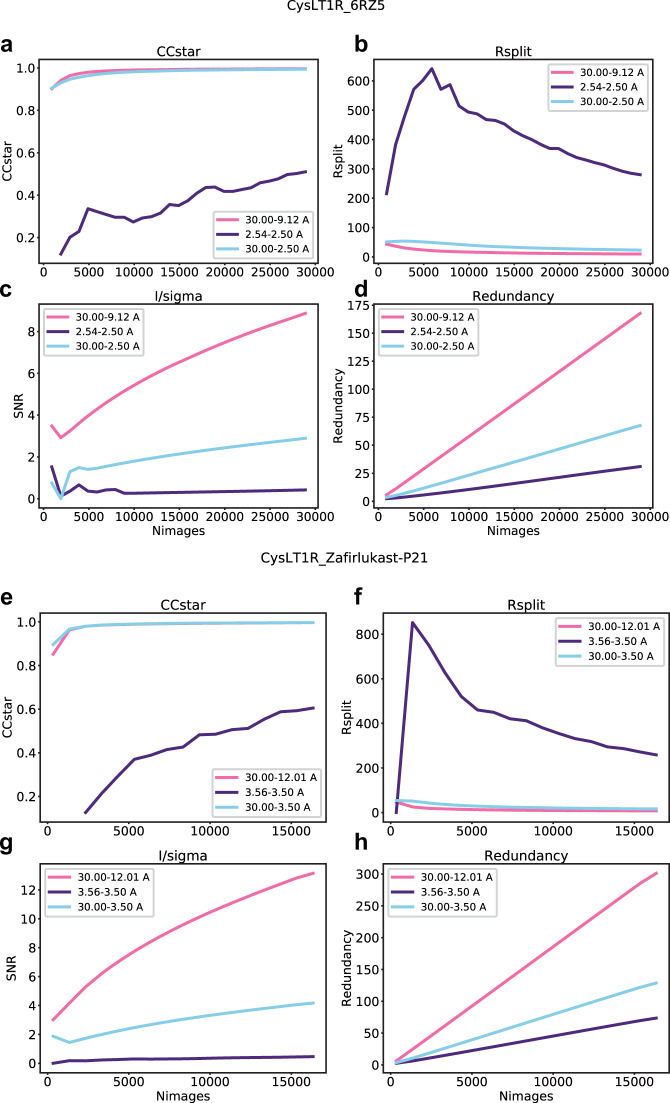
Improvements in SFX data processing statistics with increasing the number of crystals. (**a**–**d**) Plots of I/σ (*a*), R_split_ (*b*), CC* (*c*) and redundancy (*d*) in three major resolution shells (low resolution, high resolution and overall) vs the number of crystals used for merging for the dataset CysLT1R_6RZ5. (**e**–**h**) The same as panels (**a**–**d**) but for the dataset CysLT1R_Zafirlukast_P21.

*CysLT1R_6RZ5*. Previously published data^28^ were processed using CrystFEL (v. 0.6.3 + 23ea03c7). For peak finding, peakfinder8 with min-snr = 4.5, threshold = 210 was used. For indexing, the following indexers were employed: felix, dirax, asdf, taketwo, mosflm-nolatt-cell, mosflm-nocell-latt, and xds (in that order), with–multi option enabled. Data were merged using process_hkl, with push-res = 1.8 and max-adu = 14,000. For reprocessing, the same parameters in CrystFEL (v. 0.8.0) were used. The final reprocessed dataset included 28,900 indexed lattices (67% of the frames selected by Cheetah). Among indexers, felix was the most successful one, providing 16,717 indexed lattices (57.8% of all indexed lattices). Improvements of R_split_, CC* and I/sigma are shown in Fig. 4a–d.

#### CysLT1R_Zafirlukast-P21

Data (previously unpublished) were processed using CrystFEL (v. 0.8.0). For peak finding, peakfinder8 with highres = 3.0, min-snr = 4.4, threshold = 20, max-res = 300 and min-res = 80 was used. For indexing, indexers dirax, taketwo, mosflm, xds, and asdf (in that order) were used. Data were merged using process_hkl, with mincc = 0.3 and push-res = 5.0. The final dataset resulted in 17,193 indexed lattices (79% of the frames selected by Cheetah). Among indexers, dirax was the most successful one, providing 14,457 indexed lattices. Improvements of Rpim, CC* and I/sigma are shown in Fig. 4e–h.

## Usage Notes

### Downloading data

The information about downloading data is shown in Table 3. A Linux script ‘download_all.sh‘, fetching all data using *curl* utility is provided on the Github gist, associated with the publication. Folder with each entry is archived in a single tar.gz file for more convenient fetching.

### Data processing assistance scripts

Here, a brief description of scripts is given. Please, find a more detailed description in the github gist (https://gist.github.com/marinegor/96102c9b7ce87509a0832649d11ba927), associated with the publication.

1. create_xscale.inp.py — a simple script to include all existing XDS_ASCIIs to XSCALE.INP
  Given the structure of folders as in data deposited in this publication, creates an input file for express.py in the csv format
2. express.py — the SWSX integration pipeline
  Given a list of folders with XDS.INP and a path to the respective data sets, the script runs XDS for all data sets in the list, optionally adding UNIT_CELL_CONSTANTS, SPACE_GROUP_NUMBER, INCLUDE_RESOLUTION_RANGE, setting SPOT_RANGE same as DATA_RANGE, and setting REFERENCE_DATA_SET. Adds MAXIMUM_NUMBER_OF_PROCESSORS and MAXIMUM_NUMBER_OF_JOBS for processing on large clusters. Runs xscale_par afterwards.
3. xdscc.py — parsing of XDSCC.LP logfile of *xdscc12* utility for rejection of minisets based on ΔCC_1/2_.
  Analyses the output of *xdscc12* utility together with the last XSCALE.INP used, providing the list of datasets with their ΔCC_1/2_ values. Saves list of those which have ΔCC_1/2_ higher than the input threshold value.
4. reject.sh — iterative “until no dataset with negative ΔCC_1/2_ are left” dataset rejection script
  Scales XDS_ASCII.HKL files in all subfolders of the current folder. Then iteratively runs ΔCC_1/2_ rejection with the given resolution range and the number of cycles. Saves all intermediate XSCALE.INP-s and XSCALE.LP-s.
5. run_crystfel.sh
  A wrapper for the *indexamajig* routine, which i) arranges all CrystFEL-related files into subfolders, ii) automatically assigns the date and time for each generated stream and respective log file, iii) links the last created stream to ‘laststream‘ link, and shuffles the input file list, so that one could quickly and reliably check the indexing rate before the indexing finishes.
6. analysis.sh

A wrapper for *process_hkl*, *partialator*, *check_hkl*, and *compare_hkl* routines, which produces an XSCALE.LP-like statistics table, counts images indexed with different indexers, produces a command-line visible histogram of the image resolution (for a simple estimation of the push-res parameter), and writes logs.

## Supplementary information

Supplementary Table 1

## Data Availability

The code used for data reprocessing (see usage notes in *Technical validation* section) is available on github gist (https://gist.github.com/marinegor/96102c9b7ce87509a0832649d11ba927). The utility xdscc12 is available through XDS-Wiki website (https://strucbio.biologie.uni-konstanz.de/xdswiki/index.php/Xdscc12). In previous publications, for SFX data processing, CrystFEL version 0.6.3 + 23ea03c7 (available on https://stash.desy.de/projects/CRYS/repos/crystfel/commits) was used. For SWSX data processing, XDS (version BUILT = 20161101 for CysLT1R_6RZ4 and BUILT = 20161205 for CysLT2R_6RZ5–9) and XSCALE (version BUILT = 20161101 for CysLT1R_6RZ4 and BUILT = 20180319 for CysLT2R_6RZ4–9) were used in the original publication, together with the “neggia” library for reading HDF5 images, as described (https://strucbio.biologie.uni-konstanz.de/xdswiki/index.php/Eiger). For data reprocessing, XDS and XSCALE version BUILT = 20190315, and CrystFEL 0.8.0, were used.
