# Author Correction: Small-wedge synchrotron and serial XFEL datasets for Cysteinyl leukotriene GPCRs

**DOI:** 10.1038/s41597-020-00759-w

**Published:** 2020-11-23

**Authors:** Egor Marin, Aleksandra Luginina, Anastasiia Gusach, Kirill Kovalev, Sergey Bukhdruker, Polina Khorn, Vitaly Polovinkin, Elizaveta Lyapina, Andrey Rogachev, Valentin Gordeliy, Alexey Mishin, Vadim Cherezov, Valentin Borshchevskiy

**Affiliations:** 1 Research Сenter for Molecular Mechanisms of Aging and Age-related Diseases, Moscow Institute of Physics and Technology, Dolgoprudny, 141701 Russia; 2 Institut de Biologie Structurale (IBS), Université Grenoble Alpes, CEA, CNRS, Grenoble, France; 3 Institute of Biological Information Processing (IBI-7: Structural Biochemistry), Forschungszentrum Jülich GmbH, 52425 Jülich, Germany; 4 Institute of Crystallography, University of Aachen (RWTH), Aachen, Germany; 5 Bridge Institute, Michelson Center for Convergent Bioscience, University of Southern California, Los Angeles, CA 90089 USA; 6 Department of Chemistry, University of Southern California, Los Angeles, CA 90089 USA; 7 JuStruct: Jülich Center for Structural Biology, Forschungszentrum Jülich GmbH, 52425 Jülich, Germany; 8 Joint Institute for Nuclear Research, Dubna, 141980 Russia; 9 ELI Beamlines, Institute of Physics, Czech Academy of Science, Na Slovance 2, 18221 Prague, Czech Republic; 10 Present Address: MRC Laboratory of Molecular Biology, Cambridge, CB2 0QH UK; 11 ESRF—The European Synchrotron, 38000 Grenoble, France

Correction to: *Scientific Data* 10.1038/s41597-020-00729-2, published online 12 November 2020

Following publication, it was found that an incorrect grant number was attributed to the Russian Foundation for Basic Research (RFBR) in the Acknowledgements section of this Data Descriptor. This has been corrected in both the HTML and PDF versions.

